# Supplementary material for: High-Resolution Bioassay Profiling with Complemented Sensitivity and Resolution for Pancreatic Lipase Inhibitor Screening
Source: Molecules. 2022 Oct 15;27(20):6923. doi: 10.3390/molecules27206923 (PMC9607159; doi:10.3390/molecules27206923)
Supplement: Supplementary file 1 [file molecules-27-06923-s001.zip › molecules-1968707-supplementary.pdf]

Supplementary Material

High-Resolution Bioassay Profiling with Complemented Sensitivity and Resolution for Pancreatic Lipase Inhibitor Screening

Jingyi Jian [1,†], Jiaming Yuan [1,†], Yu Fan [1,†], Jincai Wang [1], Tingting Zhang [1], Jeroen Kool [2] and Zhengjin Jiang [1,*]

1 Institute of Traditional Chinese Medicine & Natural Products, College of Pharmacy/Guangdong Province Key Laboratory of Pharmacodynamic Constituents of TCM and New Drugs Research/International Cooperative Laboratory of Traditional Chinese Medicine Modernization and Innovative Drug Development of Ministry of Education (MOE) of China, Jinan University, Guangzhou 510632, China

2 Division of BioAnalytical Chemistry, Amsterdam Institute of Molecules, Medicines and Systems, Vrije Universiteit Amsterdam, 1081 HV Amsterdam, The Netherlands

* Correspondence: jzjjackson@hotmail.com

† These authors contributed equally to this work.

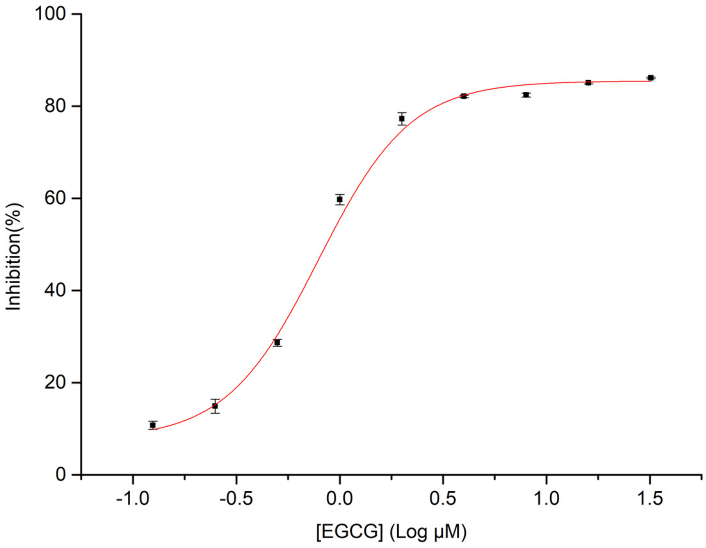

Figure S1. Dose-response curve of EGCG (IC_{50} of EGCG: 0.97 μM; R^2: 0.993).

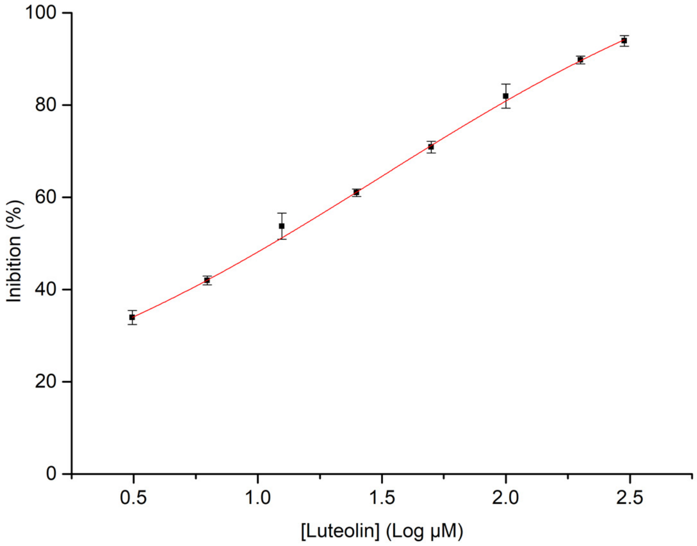

Figure S2. Dose-response curve of luteolin (IC_{50} of luteolin: 30.57 µM; R^2: 0.999).
